# The challenges ahead for patients with feeding and eating disorders during the COVID-19 pandemic

**DOI:** 10.1186/s40337-020-00322-3

**Published:** 2020-09-07

**Authors:** Mohsen Khosravi

**Affiliations:** Department of Psychiatry and Clinical Psychology, Zahedan University of Medical Sciences, Zahedan, Iran

**Keywords:** Coronavirus, Feeding and eating disorders, Pandemics, Patients

## Abstract

The 2019 coronavirus disease (COVID-19) became a global pandemic only a few months after it emerged in China. Besides, this pandemic imposed adverse consequences on public health, particularly on the mental health status of individuals with serious mental illness. However, the adverse effects of the COVID-19 pandemic on the patients with feeding and eating disorders (FEDs) are at this stage unclear. In this letter, the author seeks to acknowledge the risks of worsening FEDs during this uncertain period.

## Main text

A public health emergency may affect both individuals and communities. Individual health, well-being, and safety may be influenced due to feelings of confusion, emotional isolation, insecurity, and stigma. Furthermore, communities are affected due to the inevitable closure of schools and workplaces, the economic loss, in addition to the increased demands for medical equipment. These effects may be reflected in a variety of emotional reactions, e.g., distress or psychiatric disorders, as well as unhealthy behaviors, e.g., maladaptive eating behaviors, in both the healthcare professionals and the general population. Research has found that emotional distress is ubiquitous in affected populations. Indeed, this finding will be echoed in the affected populations in the 2019 coronavirus disease (COVID-19) pandemic [1]. Despite a lack of definitive studies to evaluate the impact of COVID-19 pandemic on mental health, survey research conducted in China has suggested moderate to severe adverse effects on the mental health in over half of participants [2].

Furthermore, considering the effects of the previous major worldwide health disasters, this pandemic is expected to increases the individuals’ stress level and lead to an increased rate of depression and anxiety [3]. Furthermore, individuals who have a previous mental disorder are at higher risk of experiencing mental health status exacerbation owing to the current pandemics [2, 4]. Thus, this pandemic is expected to worsen the severity of the feeding and eating disorders (FEDs) symptomatology due to several reasons, some of which are discussed below:

### Food insecurity in the time of COVID-19

Food insecurity is defined as the food intake disruption or alteration of eating patterns caused by a shortage of money or other resources. Several factors, such as employment, income, ethnicity/race, and disability, affect food insecurity. Food insecurity risk is intensified by a shortage of money or lack money [5]. The COVID-19 pandemic can potentially aggravate FEDs and the associated mental health symptoms because of the intensified economic limitations resulting in food insecurity (that is, restricted food access caused by economic problems). Many studies have suggested an association between food insecurity and binge eating, bulimia nervosa, and obesity [6–8]. This may be because people have insufficient funds to purchase sufficient food. These episodes of restriction will increase the risk of binge eating through food cravings as well as biological effects of starvation. Another explanation, which is maybe complementary, is the stress caused by economic strains may, in turn, promote binge eating.

Furthermore, it can be stated that the cost of binge eating or the adverse effects of experiencing FEDs on educational accomplishment or employment visions causes economic disadvantage and food insecurity. Additionally, it is established that in families with food shortage, the family members may grow a sense of shame regarding their appetite or a sense of guilt about eating without concerning to take food from others in the family. These guilt feelings may be worsened in underweight patients who are encouraged to eat more than their family members to support weight restoration [9].

### Stressful effects of daily news

FEDs may be initiated due to psychosocial stress. Studies have suggested that some individuals use disturbed eating behaviors, including binging and purging, for coping with their negative emotions. According to the previous studies on the clinical populations, an association has been found between the stressful events and the worsening of disturbed eating behaviors [10]. These exacerbations may be caused due to several reasons, such as the stress-triggering impacts of the daily reports of new cases and mortality rate of the disease (some mental health professionals call it “headline stress disorder” [11]; worrying about infection and death of their family members [12]; the inescapable media coverage on the grocery shopping, food safety, food shortages threats, and “how to control emotional eating”; or the focus of some online contents regarding the pandemic on “how to appear perfect on a webcam”) as well as at-home workout challenges can involuntarily strengthen the eating-disorder behaviors and cognitions [9].

### Concerns about health and fitness during the quarantine

Concerns regarding the health and fitness in the quarantine may contribute to FEDs development in prone individuals. Moreover, additional risk factors may increase the risk of FEDs development. These risk factors include spending more time on social media, and the destructive impact of the thin ideal objectification. Loneliness and isolation are common causes of anorexia nervosa, which may be intensified due to the imposed quarantine. The emotional imbalance may cause FEDs symptoms (i.e., episodes of binge eating episodes followed by purging behaviors) [13], while increases external control may reduce the food intake [14]. Family members’ maladaptive emotional reactions caused by the quarantine stress may result in aggression and/or splitting and fragmentation [15]. Moreover, the orders of staying at home and the limited food selection choices in stores may make it look rational for some individuals to skip meals or restrict their calorie intake, which will subsequently worsen the preexisting restrictive tendencies [9].

### Disruption of access to professional support

Obeying physical distancing as well as the global mantra of staying at home has challenged the face-to-face hospital programs [16]. The current disrupted access to reliable cares may attract FEDs patients to unreliable sources, e.g., social media (i.e., Whatsapp, Instagram, Telegram, etc.) [17]. Moreover, professionals’ time limitation for the preparation of FEDs patients and their families for the treatment change was associated with an intensified fear and feeling of losing control, which was already felt with the COVID-19 spread. Online therapy is available for some patients, though experts agree that in the case of absent face-to-face accountability (i.e., absence of weigh-ins), increased self-management is vital for FEDs patients [15]. To summarize, the COVID-19 pandemic probably leads to an exacerbation of the mental health of patients with FEDs owing to the logistic challenges caused by safety protocols developed for COVID-19 [9].

To conclude, taking into account the confinement and the uncertainty-caused distress in the FEDs patients who are at higher physical risks (including frailty caused by anorexia nervosa, electrolyte imbalance due to bulimia nervosa, and the cardiovascular risk caused by binge eating) as well as reduced access to the usual treatments [15], it is crucial to develop evidenced-based practice. This is in order to diminish the adverse consequences of COVID-19 and learn from this unpredictable crisis for future benefits.

## Data Availability

N/a

## Abbreviations

COVID-19: The 2019 coronavirus disease
FEDs: Feeding and eating disorders
